# Expression-Based Cell Lineage Analysis in *Drosophila* Through a Course-Based Research Experience for Early Undergraduates

**DOI:** 10.1534/g3.119.400541

**Published:** 2019-09-19

**Authors:** John M. Olson, Cory J. Evans, Kathy T. Ngo, Hee Jong Kim, Joseph Duy Nguyen, Kayla G. H. Gurley, Truc Ta, Vijay Patel, Lisa Han, Khoa T. Truong-N, Letty Liang, Maggie K. Chu, Hiu Lam, Hannah G. Ahn, Abhik Kumar Banerjee, In Young Choi, Ross G. Kelley, Naseem Moridzadeh, Awais M. Khan, Omair Khan, Szuyao Lee, Elizabeth B. Johnson, Annie Tigranyan, Jay Wang, Anand D. Gandhi, Manish M. Padhiar, Joseph Hargan Calvopina, Kirandeep Sumra, Kristy Ou, Jessie C. Wu, Joseph N. Dickan, Sabrena M. Ahmadi, Donald N. Allen, Van Thanh Mai, Saif Ansari, George Yeh, Earl Yoon, Kimberly Gon, John Y. Yu, Johnny He, Jesse M. Zaretsky, Noemi E. Lee, Edward Kuoy, Alexander N. Patananan, Daniel Sitz, PhuongThao Tran, Minh-Tu Do, Samira J. Akhave, Silverio D. Alvarez, Bobby Asem, Neda Asem, Nicole A. Azarian, Arezou Babaesfahani, Ahmad Bahrami, Manjeet Bhamra, Ragini Bhargava, Rakesh Bhatia, Subir Bhatia, Nicholas Bumacod, Jonathan J. Caine, Thomas A. Caldwell, Nicole A. Calica, Elise M. Calonico, Carman Chan, Helen H.-L. Chan, Albert Chang, Chiaen Chang, Daniel Chang, Jennifer S. Chang, Nauman Charania, Jasmine Y. Chen, Kevin Chen, Lu Chen, Yuyu Chen, Derek J. Cheung, Jesse J. Cheung, Jessica J. Chew, Nicole B. Chew, Cheng-An Tony Chien, Alana M. Chin, Chee Jia Chin, Youngho Cho, Man Ting Chou, Ke-Huan K. Chow, Carolyn Chu, Derrick M. Chu, Virginia Chu, Katherine Chuang, Arunit Singh Chugh, Mark R. Cubberly, Michael Guillermo Daniel, Sangita Datta, Raj Dhaliwal, Jenny Dinh, Dhaval Dixit, Emmylou Dowling, Melinda Feng, Christopher M. From, Daisuke Furukawa, Himaja Gaddipati, Lilit Gevorgyan, Zunera Ghaznavi, Tulika Ghosh, Jaskaran Gill, David J. Groves, Kalkidan K. Gurara, Ali R. Haghighi, Alexandra L. Havard, Nasser Heyrani, Tanya Hioe, Kirim Hong, Justin J. Houman, Molly Howland, Elaine L. Hsia, Justin Hsueh, Stacy Hu, Andrew J. Huang, Jasmine C. Huynh, Jenny Huynh, Chris Iwuchukwu, Michael J. Jang, An An Jiang, Simran Kahlon, Pei-Yun Kao, Manpreet Kaur, Matthew G. Keehn, Elizabeth J. Kim, Hannah Kim, Michelle J. Kim, Shawn J. Kim, Aleksandar Kitich, Ross A. Kornberg, Nicholas G. Kouzelos, Jane Kuon, Bryan Lau, Roger K. Lau, Rona Law, Huy D. Le, Rachael Le, Carrou Lee, Christina Lee, Grace E. Lee, Kenny Lee, Michelle J. Lee, Regina V. Lee, Sean H. K. Lee, Sung Kyu Lee, Sung-Ling D. Lee, Yong Jun Lee, Megan J. Leong, David M. Li, Hao Li, Xingfu Liang, Eric Lin, Michelle M. Lin, Peter Lin, Tiffany Lin, Stacey Lu, Serena S. Luong, Jessica S. Ma, Li Ma, Justin N. Maghen, Sravya Mallam, Shivtaj Mann, Jason H. Melehani, Ryan C. Miller, Nitish Mittal, Carmel M. Moazez, Susie Moon, Rameen Moridzadeh, Kaley Ngo, Hanh H. Nguyen, Kambria Nguyen, Thien H. Nguyen, Angela W. Nieh, Isabella Niu, Seo-Kyung Oh, Jessica R. Ong, Randi K. Oyama, Joseph Park, Yaelim A. Park, Kimberly A. Passmore, Ami Patel, Amy A. Patel, Dhruv Patel, Tirth Patel, Katherine E. Peterson, An Huynh Pham, Steven V. Pham, Melissa E. Phuphanich, Neil D. Poria, Alexandra Pourzia, Victoria Ragland, Riki D. Ranat, Cameron M. Rice, David Roh, Solomon Rojhani, Lili Sadri, Agafe Saguros, Zainab Saifee, Manjot Sandhu, Brooke Scruggs, Lisa M. Scully, Vanessa Shih, Brian A. Shin, Tamir Sholklapper, Harnek Singh, Sumedha Singh, Sondra L. Snyder, Katelyn F. Sobotka, Sae Ho Song, Siddharth Sukumar, Halley C. Sullivan, Mark Sy, Hande Tan, Sara K. Taylor, Shivani K. Thaker, Tulsi Thakore, Gregory E. Tong, Jacinda N. Tran, Jonathan Tran, Tuan D. Tran, Vivi Tran, Cindy L. Trang, Hung G. Trinh, Peter Trinh, Han-Ching H. Tseng, Ted T. Uotani, Akram V. Uraizee, Kent K. T. Vu, Kevin K. T. Vu, Komal Wadhwani, Paluk K. Walia, Rebecca S. Wang, Shuo Wang, Stephanie J. Wang, Danica D. Wiredja, Andrew L. Wong, Daniel Wu, Xi Xue, Griselda Yanez, Yung-Hsuan Yang, Zhong Ye, Victor W. Yee, Cynthia Yeh, Yue Zhao, Xin Zheng, Anke Ziegenbalg, Jon Alkali, Ida Azizkhanian, Akash Bhakta, Luke Berry, Ryen Castillo, Sonja Darwish, Holly Dickinson, Ritika Dutta, Rahul Kumar Ghosh, Riley Guerin, Jonathan Hofman, Garrick Iwamoto, Sarah Kang, Andrew Kim, Brian Kim, Hanwool Kim, Kristine Kim, Suji Kim, Julie Ko, Michael Koenig, Alejandro LaRiviere, Clifton Lee, Jiwon Lee, Brandon Lung, Max Mittelman, Mark Murata, Yujin Park, Daniel Rothberg, Ben Sprung-Keyser, Kunal Thaker, Vivian Yip, Paul Picard, Francie Diep, Nikki Villarasa, Volker Hartenstein, Casey Shapiro, Marc Levis-Fitzgerald, Leslie Jaworski, David Loppato, Ira E. Clark, Utpal Banerjee

**Affiliations:** * Department of Molecular, Cell and Developmental Biology, University of California, Los Angeles, Los Angeles, CA 90095; † Biomedical Research Minor, University of California, Los Angeles; ‡ Loyola High School, Los Angeles, CA 90006; § Center for the Advancement of Teaching, University of California, Los Angeles, and; ** Department of Psychology, Grinnell College, Grinnell, IA 50112

**Keywords:** G-TRACE, gene expression, education, STEM, CURE

## Abstract

A variety of genetic techniques have been devised to determine cell lineage relationships during tissue development. Some of these systems monitor cell lineages spatially and/or temporally without regard to gene expression by the cells, whereas others correlate gene expression with the lineage under study. The GAL4 Technique for Real-time and Clonal Expression (G-TRACE) system allows for rapid, fluorescent protein-based visualization of both current and past GAL4 expression patterns and is therefore amenable to genome-wide expression-based lineage screens. Here we describe the results from such a screen, performed by undergraduate students of the University of California, Los Angeles (UCLA) Undergraduate Research Consortium for Functional Genomics (URCFG) and high school summer scholars as part of a discovery-based education program. The results of the screen, which reveal novel expression-based lineage patterns within the brain, the imaginal disc epithelia, and the hematopoietic lymph gland, have been compiled into the G-TRACE Expression Database (GED), an online resource for use by the *Drosophila* research community. The impact of this discovery-based research experience on student learning gains was assessed independently and shown to be greater than that of similar programs conducted elsewhere. Furthermore, students participating in the URCFG showed considerably higher STEM retention rates than UCLA STEM students that did not participate in the URCFG, as well as STEM students nationwide.

Cell lineage analysis within tissues has contributed significantly to our understanding of the morphogenetic events that occur during the development of multicellular organisms. In *Drosophila* in particular, a vast repertoire of powerful genetic tools has been created for and utilized in such developmental analyses (Brand and Perrimon 1993; Struhl and Basler 1993; Harrison and Perrimon 1993; Pignoni and Zipursky 1997; Ito *et al.* 1997; Lee *et al.* 1999; Weigmann and Cohen 1999; Osterwalder *et al.* 2001; McGuire *et al.* 2003; Jung *et al.* 2005; Yu *et al.* 2009; Griffin *et al.* 2009, 2014; Evans *et al.* 2009; Hampel *et al.* 2011; Hadjieconomou *et al.* 2011; del Valle Rodriguez *et al.* 2011; Worley *et al.* 2013; Kanca *et al.* 2014). One such tool is the G-TRACE system, which was developed by the UCLA Undergraduate Research Consortium for Functional Genomics (URCFG) for high-throughput gene expression-based lineage analysis (Evans *et al.* 2009). The URCFG, developed in 2003 as part of our HHMI Professors program, involves undergraduate students in actual scientific research at an early stage of their academic careers. URCFG students, primarily first- and second-year undergraduates, conduct original laboratory research in the context of a 10-week, academic-year course, *Life Sciences 10H: Research Training in Genes*, *Genetics and Genomics* (now listed as *Biomedical Research 10H*), the pedagogical details of which have been described elsewhere (Chen *et al.* 2005; Call *et al.* 2007). Previous accomplishments by URCFG undergraduate researchers include the analysis of the effect of 2,100 lethal mutations on the development of the adult *Drosophila* eye (Chen *et al.* 2005; Call *et al.* 2007).

Here, URCFG students used the G-TRACE system to analyze cell populations as defined by the activity of endogenous gene enhancer elements. One way to monitor gene enhancer activity in *Drosophila* is through the use of the well-established, bipartite GAL4/UAS transcriptional control system (adapted from yeast; Elliott and Brand 2008) as reporter. In this system, unique *Drosophila* enhancer elements control the expression of the GAL4 transcriptional activator, which in turn activates the expression of any gene placed under the control of the yeast-specific UAS enhancer element. The G-TRACE system reports current or “real-time” GAL4 activity through the expression of the red fluorescent protein (RFP) DsRed (*UAS-DsRed*), while also identifying all daughter cell progeny from such cells (the GAL4-positive cell lineage) through the expression of enhanced green fluorescent protein (GFP) (see Figure 1 and Evans *et al.* 2009 for a full description of the G-TRACE system). Thus, G-TRACE analysis can reveal dynamic gene enhancer activity by comparing current GAL4 activity (RFP) and lineage-traced GAL4 activity (GFP), which may have similar, overlapping or widely different patterns of expression at the point of analysis. The real-time and lineage expression patterns associated with any GAL4-expressing line can easily be assessed by simply crossing with the G-TRACE test stock and examining RFP and GFP fluorescence in the developing progeny.

**Figure 1:**
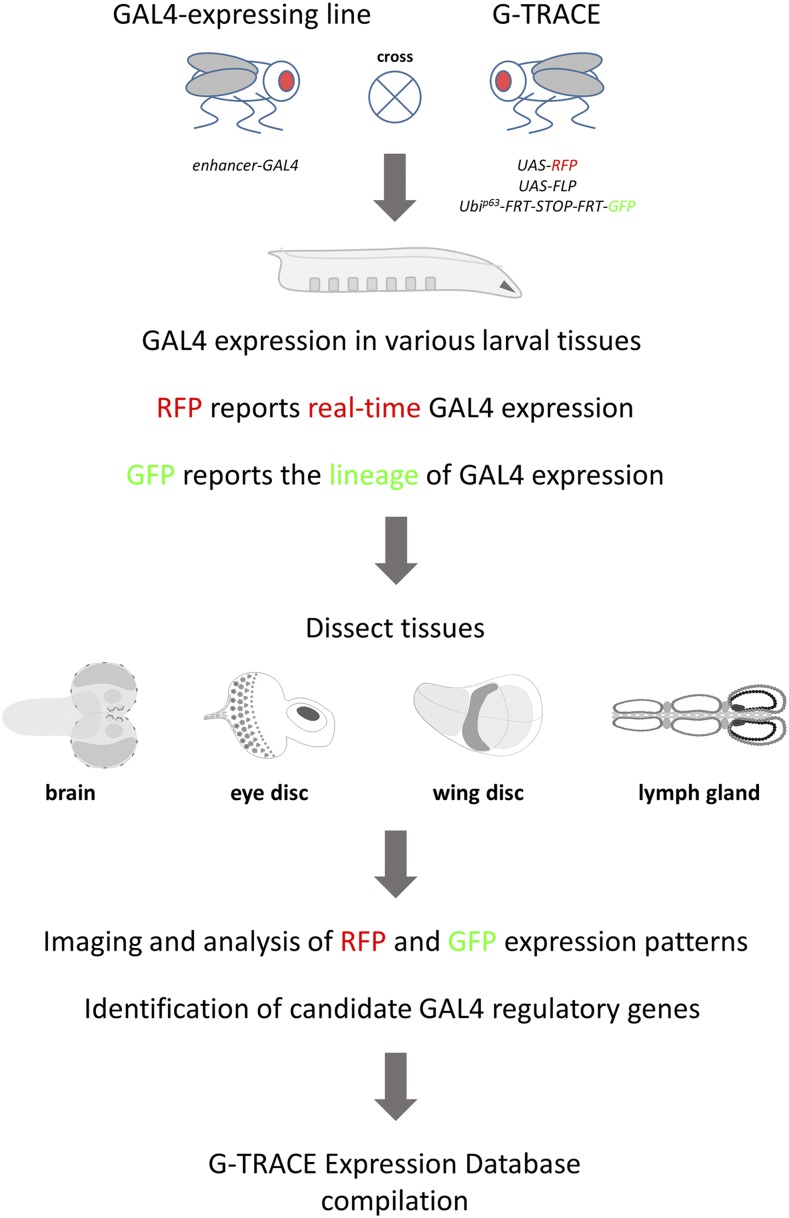
Overview of the G-TRACE screening strategy. Transgenic *Drosophila* lines expressing GAL4 (*enhancer-GAL4* lines; *P{GawB}* NP lines) are crossed to the G-TRACE screening stock. Progeny larvae will express GAL4 protein in various tissues, dependent upon enhancer activity, which will be reported by the expression of RFP (DsRed.T4). The GAL4-expressing cell will also initiate the cell lineage marker GFP, which will be expressed perpetually by all subsequent daughter cells (see Evans *et al.*, 2009 for a complete description of the G-TRACE labeling mechanism). Wandering third-instar larvae from such crosses are collected, followed by the dissection of the brain, eye and wing imaginal discs, and the lymph gland (the hematopoietic organ). These tissues are subsequently mounted on glass slides for imaging by fluorescence microscopy, followed by analysis of RFP and GFP expression patterns. Using basic bioinformatics approaches, endogenous genes proximal to the GAL4 insertion site are identified. For each GAL4 line, representative fluorescence microscopy images, RFP/GFP expression data, and associated candidate regulatory gene information are assembled into the G-TRACE Expression Database (GED), a searchable, online database.

Using the G-TRACE analysis system, 245 URCFG students and 31 high school summer scholars analyzed hundreds of unique GAL4-expressing lines. Each line was crossed to the G-TRACE reporter stock and the subsequent real-time (RFP) and lineage (GFP) expression patterns arising in four developing larval tissues (the brain, eye and wing discs, and the lymph gland) were examined. Several students also participated during multiple academic quarters to verify prior work. In this paper, we highlight some of the discoveries made by URCFG students and introduce the G-TRACE Expression Database (GED; www.urcfg.ucla.edu), which reports data associated with 563 different GAL4 lines. We expect that the GED will be a valuable resource for members of the *Drosophila* community interested in the development of the larval brain, eye and wing discs, and lymph gland, as well as the identity of genes and GAL4 lines expressed within these tissues.

From an educational perspective, the G-TRACE URCFG project has provided an effective means for direct engagement of a large number of early undergraduate (as well as high school) students in the process of scientific discovery. The President’s Council of Advisors on Science and Technology (PCAST 2012) has argued that early engagement in inquiry-based learning encourages students to persist in STEM (science, technology, engineering, and mathematics) disciplines. Consistent with this idea, we find that students who participated in this and prior URCFG research programs earned STEM degrees at a higher rate than those that did not participate in the URCFG.

## Materials and Methods

### Genetics, tissue processing, and fluorescence microscopy

All NP line GAL4 drivers used in this study were obtained from the KYOTO Stock Center (DGGR), Kyoto Institute of Technology, Japan. The full genotype of the G-TRACE line used for the analysis is: *UAS-Flp*, *UAS-DsRed*, *ubi-p63E-FRT-stop-FRT-nEGFP/CyO* (as described in Evans *et al.* 2009; Bloomington *Drosophila* Stock 28280). Crosses were grown at 25° unless larval lethality was observed, possibly due to high-level DsRed expression is sensitive tissues (Barolo *et al.* 2004; Strack *et al.* 2008). In such cases, crosses were alternatively grown at 22° to reduce GAL4 activity. Wandering third instar larvae were selected and tissues were dissected using standard procedures in 1X Phosphate Buffered Saline (PBS, pH =7.4). Dissected tissues were fixed in 3.7% formaldehyde/1XPBS for 20 min at room temperature. Samples were briefly washed in 1XPBS containing DAPI (1/1000; Invitrogen) to label DNA for fluorescent microscopy. Samples were mounted in 80% glycerol and imaged using a Zeiss AxioImager.Z1 microscope equipped with the ApoTome acquisition system. Images were processed and Z-projections were made using Zeiss AxioVision LE 4.4 software. All cells expressing DsRed should also express GFP; however examples are often observed in which RFP is expressed by cells without apparent co-expression of GFP. This phenomenon is related to GAL4 expression level, threshold effects associated with FLP/FRT-mediated removal of the transcriptional STOP cassette, and the cell type-specific activity of the *Ubi-p63* promoter, which is discussed in Evans *et al.* 2009. Expression data for each NP line was verified independently.

### G-TRACE Expression Scoring

Expression of G-TRACE RFP and GFP patterns within the third instar brain, eye and wing imaginal discs, and the lymph gland were scored based upon spatial overlap with established tissue regions or cell types. Prior to scoring images, students were informed about different relevant cell types and tissue regions for each dissected tissue as part of the course pedagogy, which included the use of schematics similar to those shown in the data figures here. For the brain, the following areas were identified: the central brain (CB, including the ventral nerve cord), mushroom body (MB) neurons, Type II lineage neurons, surface glia (SG), and the optic lobe (OL), including medulla primordia (MP) and lobula primordia (LOP) subregions. For the eye-antennal imaginal disc, expression was scored as ubiquitous or within the eye disc proper, photoreceptors (PR), eye glia, antenna, arista, or the peripodial membrane (PM). For the wing imaginal disc, expression was scored as ubiquitous or within the notum, hinge, pouch, anterior-posterior (A/P) or dorsal-ventral (D/V) boundary, or peripodial membrane (PM) and trachea. In the lymph gland, expression was scored as ubiquitous or within the primary lobe (PL), including the cortical zone (CZ) and posterior signaling center (PSC) subregions, secondary lobes (SL) or tertiary lobes (TL), the dorsal vessel (DV), or pericardial cells (PC).

### Identification of candidate genes controlling GAL4 expression

Using GAL4-line stock numbers, students searched the online database FlyBase (Attrill *et al.* 2016) and retrieved genotypes that were hyperlinked to individual pages within the GAL4 Enhancer Trap Database (GETDB, now integrated into the *Drosophila* Genomics and Genetic Resources/Kyoto Stock Center). For each GAL4 transgenic line (which are transposable *P*-element-based *P{GawB}* insertions), students copied available flanking DNA sequence, and used the FlyBase BLAST feature to identify the genomic position of the transgene insertion. Then, using the FlyBase GBrowse genome browser feature, students identified the three closest genes, up to 50 kilobase pairs (kbp) away (based upon FlyBase annotation release 5.3 or earlier). The identification of *GAL4*-proximal genes was repeated *en masse* (by instructor CJE) using unique transposable element insertion (FBti) numbers for each NP line and the FlyBase FeatureMapper tool to query an updated FlyBase genome annotation release (6.10) for genes within a defined 2 kbp distance upstream and downstream of the insertion site. The 2 kbp distance was selected as a reasonable cutoff since it has been previously shown that 70% of *P*-element inserts are located within 0.5 kbp of gene promoters (Spradling *et al.* 2011). The associated gene numbers described here reflect the updated list of candidate genes; however the original student-derived gene associations are available at the GED.

### Development of the G-TRACE Expression Database (GED)

To present G-TRACE expression pattern scores and images for each GAL4-expressing NP line, an internet-accessible database was constructed that is searchable by NP line number or gene symbol and is filterable in large-scale by expression within the brain, eye and wing imaginal discs, and the lymph gland. The GED uses the AngularJS 1.X (Google, Inc.) single-page website framework to coordinate overall functionality. Ajax-based non-refresh pagination along with the ng-Table plugin (Vitalii Savchuk) is used to handle data sorting, filtering, and presentation. To increase data retrieval speed and minimize browser function, the complete GED website, including dataset retrieval via Ajax, is designed to perform solely on the client-side once the website is loaded from the web server. Data presentation is enhanced by color-coding RFP and GFP expression scores and by using a click-and-enlarge function for images, accomplished through customizing ng-Table with in-house AngularJS-based scripts. The website front-end was wrapped with Bootstrap theme components (Twitter, Inc.) using figures/images generated in-house.

### Reagent and Data Availability

G-TRACE lines are available from the Bloomington *Drosophila* Stock Center (Bloomington, IN), and NP *GAL4* enhancer trap lines are available from the Kyoto Stock Center (Japan). G-TRACE expression data for this analysis is available to browse and search online at the G-TRACE Expression Database (GED; www.urcfg.ucla.edu). Supplementary Table S1 contains searchable RFP and GFP scoring data for the 563 GAL4 lines reported in the GED. Table S2 lists candidate regulatory genes for the 563 GAL4 lines reported in the GED, and Table S3 specifically lists candidate regulatory genes for GAL4 lines with expression in the brain LOP. All supplemental tables are in Microsoft Excel (.xlsx) format and have been uploaded to FigShare. UCLA STEM retention data were obtained under UCLA IRB#16-001388. Supplemental material available at FigShare: https://doi.org/10.25387/g3.9732146.

## Results

### G-TRACE screening by student researchers

**Figure 2:**
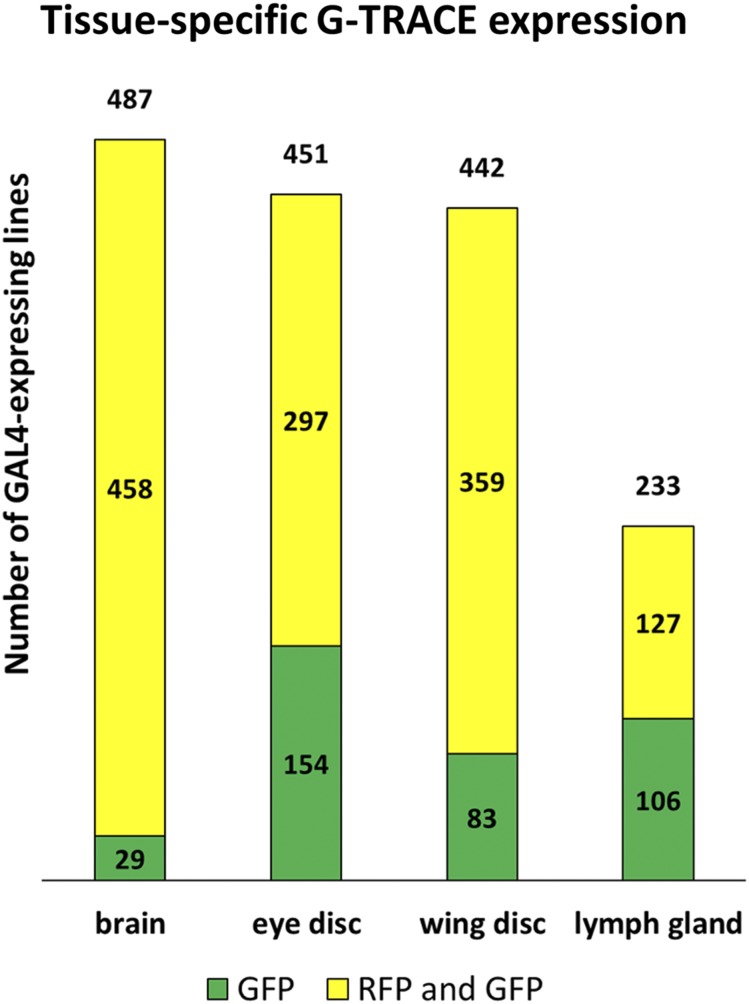
Incidence of GAL4 activity within the larval brain, eye and wing imaginal discs, and lymph gland. Bar graphs demonstrating the total number of GAL4-expressing lines identified per tissue (number above the bar, out of 563 screened) as well as the subset exhibiting either combined RFP and GFP expression (yellow) or GFP expression alone (green).

**Figure 3:**
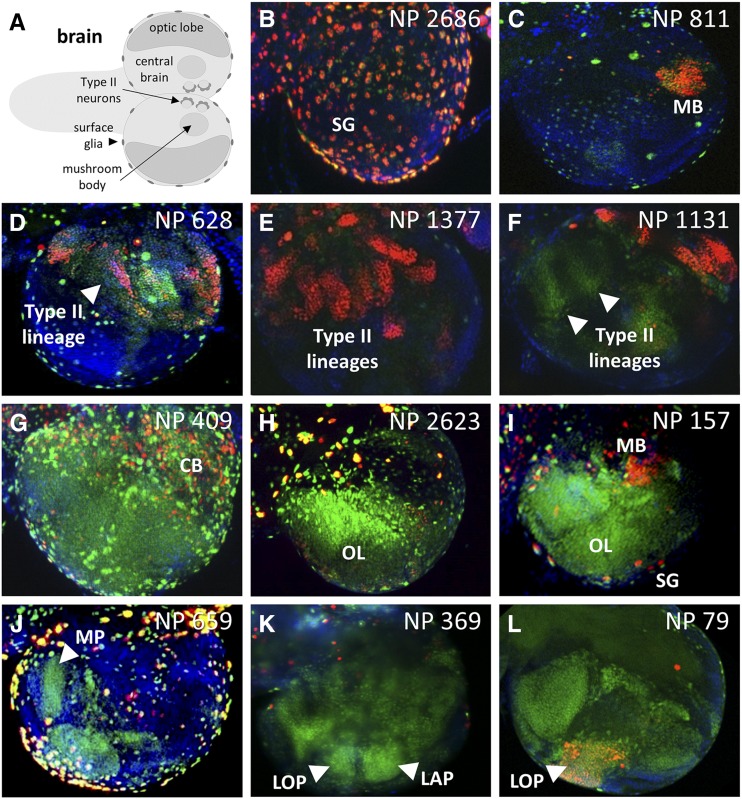
Select GAL4-expressing lines with complex G-TRACE patterns in the brain. A) Schematic of the third instar larval brain showing the primary structures identified during screening. B-L) Fluorescence microscopy images showing various patterns of real-time GAL4 activity (RFP, red) and associated cell lineages (GFP, green) within the third instar larval brain. The corresponding NP line identifier is shown in the upper right corner of each image. For all images, DNA is shown in blue (DAPI staining). Surface glia (SG); mushroom body (MB); central brain (CB); optic lobe (OL); medulla primordia (MP); lobula primordia (LOP); lamina primordia (LAP).

**Figure 4:**
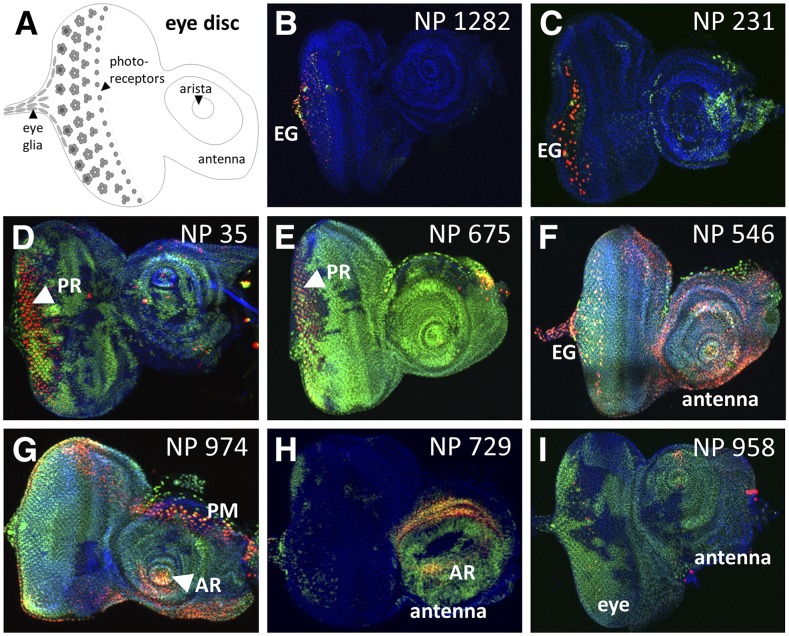
Select GAL4-expressing lines with complex G-TRACE patterns in the eye disc. A) Schematic of the third instar larval eye disc showing the primary structures identified during screening. B-I) Fluorescence microscopy images showing various patterns of real-time GAL4 activity (RFP, red) and associated cell lineages (GFP, green) within the third instar larval eye disc. The corresponding NP line identifier is shown in the upper right corner of each image. For all images, DNA is shown in blue (DAPI staining). Eye glia (EG); photoreceptors (PR); arista (AR); peripodial membrane (PM).

**Figure 5:**
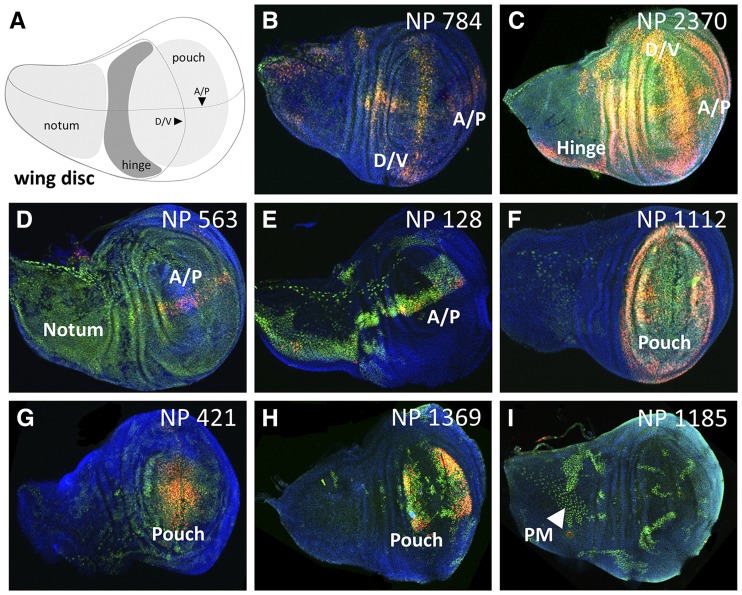
Select GAL4-expressing lines with complex G-TRACE patterns in the wing disc. A) Schematic of the third instar larval wing disc showing the primary structures identified during screening. B-I) Fluorescence microscopy images showing various patterns of real-time GAL4 activity (RFP, red) and associated cell lineages (GFP, green) within the third instar larval wing disc. The corresponding NP line identifier is shown in the upper right corner of each image. For all images, DNA is shown in blue (DAPI staining). Dorsal/ventral boundary (D/V); anterior/posterior boundary (A/P); peripodial membrane (PM).

**Figure 6:**
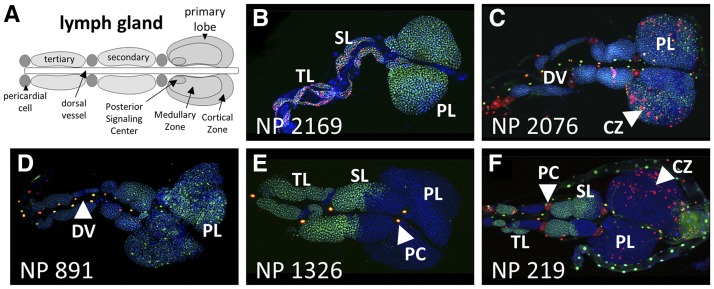
Select GAL4-expressing lines with complex G-TRACE patterns in the lymph gland. A) Schematic of the third instar larval lymph gland showing the primary structures identified during screening. B-F) Fluorescence microscopy images showing various patterns of real-time GAL4 activity (RFP, red) and associated cell lineages (GFP, green) within the third instar larval lymph gland. The corresponding NP line identifier is shown in the bottom left corner of each image. For all images, DNA is shown in blue (DAPI staining). Primary lobes (PL); secondary and tertiary lobes (SL, TL); pericardial cell (PC); Cortical Zone (CZ); dorsal vessel (DV); posterior signaling center (PSC).

For the initiation of G-TRACE patterns within developing larval tissues, students (primarily first- and second-year undergraduates) utilized transgenic GAL4-expressing lines (Nippon Project or NP lines) previously generated as part of the GAL4 Enhancer Trap Database project (GETDB; Hayashi *et al.* 2002). Each NP line represents a GAL4 “enhancer trap”, where the GAL4 gene (located within a transposable element) is inserted into the *Drosophila* genome at a unique position and is then expressed under the control of nearby endogenous enhancer elements. These GAL4-expressing lines were crossed to the G-TRACE stock, and developing brains, eye and wing imaginal discs (primordia for adult eyes and wings, respectively), and lymph glands (hematopoietic organs) from third-instar progeny larvae were dissected by students for analysis by fluorescence microscopy (Figure 1). Each student imaged and analyzed G-TRACE patterns associated with several GAL4-expressing lines (usually seven or more), and each GAL4-expressing line was analyzed by at least two students working separately, using a larval sample size (n = 10) large enough to ensure the reproducibility of the observed expression patterns. A conservative estimate of the number of images collected by students during the course of this project exceeds 50,000 images. The resulting G-TRACE RFP and GFP expression patterns were scored based upon overlap with or proximity to well-established, easily recognizable morphological structures or spatial features within the respective tissues (see Figures 2, 3, 4, 5, and 6, Table S1, and Materials and Methods).

A key feature of the GETDB collection is that the GAL4 insertion point of each NP line has been mapped to a specific location within the genome. For each of their assigned NP lines, URCFG students utilized genomic DNA sequence flanking each GAL4 transgene insertion site (available online through GETDB/DGGR, Japan; Hayashi *et al.* 2002) to query the *Drosophila* genome database (FlyBase BLAST) and find nearby endogenous genes that may be controlling GAL4 expression. Of the 563 different GAL4 lines, the transgene insertion site for 394 lines was located within one or more genes (448 genes total; FlyBase release 6.10). Analysis of genes located within 2 kilobase pairs (kbp) on either side of each insert site, which was particularly important for the 169 lines with GAL4 transgene insertions located within intergenic areas of the chromosome, identified an additional 583 candidate regulatory genes (287 upstream and 296 downstream). Thus, for the complete set of 563 GAL4 lines, a total of 1,031 candidate regulatory genes were identified, of which 416 represent previously uncharacterized genes (Table S2).

The collective results of this developmental GAL4 expression screen have been assembled into the G-TRACE Expression Database (GED), a searchable online resource for the *Drosophila* research community and beyond. For each NP line, the RFP and GFP expression patterns within the four analyzed larval tissues are reported, along with representative microscopic images and candidate regulatory gene information. The GED will be useful to a variety of researchers, particularly those interested in genetic control of the development of the larval brain, eye and wing imaginal discs, and the lymph gland. Others may be interested in identifying GAL4 lines based upon expression patterns only, simply for use as expression tools within these tissues. Lastly, there may also be researchers that are interested in identifying gene-associated GAL4 lines for use in other tissues not analyzed here.

### GAL4-dependent G-TRACE patterns in four larval tissues with select examples

Of the 563 NP lines screened using G-TRACE, 537 lines (95%) exhibited GAL4 activity in one or more tissues, while 194 lines (34%) were expressed in all four tissues. Within specific tissues, 487 lines (87%) exhibited GAL4 activity in the brain, 451 lines (80%) exhibited GAL4 activity in the eye, 442 lines (79%) exhibited GAL4 activity in the wing, and 233 lines (41%) exhibited GAL4 activity in the lymph gland (Figure 2), with 47 lines (8%) being specific to one tissue. This analysis using G-TRACE uniquely identified a large number of GAL4 lines (278) that exhibit lineage marking (GFP expression) within one or more tissues but lack any real-time GAL4 activity (RFP expression) in the late third instar, the time of examination. As previously demonstrated (Evans *et al.* 2009), such patterns reflect GAL4 activity that is restricted to early stages of development.

In the larval brain (Figure 3), a variety of developmental G-TRACE expression patterns were observed that often coincided with defined structures or cell populations. For example, a large number of lines (168) exhibited GAL4 activity in the central brain (CB), including 84 with reporter expression in the mushroom body and 30 with reporter expression within Type II neuroblast lineages. The optic lobe (OL) of the brain was also frequently associated with GAL4 activity. Within the developing optic lobe exist the medulla primordia (MP), the lamina primordia (LAP), and the lobula primordia (LOP), the last of which is the least understood developmentally due to a paucity of markers. Here, the G-TRACE system identified several GAL4 lines marking each of these optic lobe structures (Figure 3), including 25 lines with distinct LOP expression (see Table S3). Interestingly, at the stage of analysis, 21 of these 25 lines exhibited only GFP expression within the LOP, indicating that GAL4 activity is restricted to LOP precursors at an earlier stage of development. Control of GAL4 expression in these 25 LOP-expressing lines was associated with 61 different candidate genes (including 21 uncharacterized CG/CR genes and 12 *mir* genes), none of which have been previously associated with optic lobe development (based upon FlyBase GO queries using controlled vocabularies). Investigators in the *Drosophila* community interested in optic lobe development will likely find these GAL4-expressing lines useful for defining LOP progenitors and derivative populations, as well as for gain- and loss-of-function genetic studies that rely on the GAL4/UAS system (*e.g.*, using RNAi).

As with the brain, many lines were identified exhibiting region-specific GAL4 activity within the eye imaginal disc (Figure 4). For example, this screen identified 77 GAL4 lines with expression within photoreceptors (PR), which is important given that photoreceptor specification and differentiation has long been a cornerstone of *Drosophila* developmental biology research. Another interesting group of lines identified was that exhibiting GAL4 activity in eye (retinal) glia, which migrate from the brain to the eye via the optic stalk in order to interact with developing photoreceptor neurons. While most of these lines (63 of 85) exhibit eye glia with active GAL4 expression (*i.e.*, eye glia that are RFP-positive), 22 lines yield eye glia with only lineage-traced GFP expression, indicating that GAL4 must have been active in these cells or in progenitors to these cells prior to migration into the eye imaginal disc. Examples from the wing imaginal discs (Figure 5) include 20 GAL4 lines exhibiting expression (primarily real-time RFP) along the dorsal/ventral (D/V) and/or anterior/posterior (A/P) boundaries, and a large collection of lines (307) that exhibited GAL4 activity in the pouch region ranging from general to specific.

The larval lymph gland (Figure 6) consists of hemocyte (blood cell)-filled primary lobes (PLs), secondary lobes (SLs), and tertiary lobes (TLs) that bilaterally flank the dorsal vessel (heart tube) near the thoracic segments. The lymph gland primary lobes are the main sites of hematopoietic differentiation during larval development (Evans *et al.* 2014), while secondary and tertiary lobes primarily hold reserve, undifferentiated hemocytes. Pericardial cells (PCs) that behave as nephrocyte-like filtering cells also flank the dorsal vessel along its length and interdigitate between lymph gland lobes. As mentioned, 233 different GAL4 lines were found to be expressed in the lymph gland (including the dorsal vessel and pericardial cells, Figure 6), either ubiquitously or restricted to a specific area such as the primary lobe Cortical Zone (CZ), which contains mature blood cells. Also identified were several lines exhibiting regional GAL4 activity within secondary and tertiary lobes (Figure 6), which is interesting given that relatively little is known about the developmental origin of these lobes from the cardiogenic mesoderm. However, the reproducible mosaic labeling of large swaths of cells, primarily by lineage-based GFP expression, is indicative of early differential GAL4 expression among progenitors of these lobes.

## Discussion

Our large-scale analysis of gene expression-based cell lineage development in *Drosophila* by undergraduates has leveraged our previously described pedagogical method for discovery-based science education within the laboratory (Chen *et al.* 2005). Here we discuss the scientific and educational goals that motivated the use of the G-TRACE analysis system by URCFG students, highlight the resulting research products generated by the URCFG students, and relate how this approach to science education impacted student learning.

The foremost scientific goal of the G-TRACE project was the identification of new GAL4-expressing lines with spatiotemporal expression patterns in the developing larval brain, eye and wing imaginal discs, and the lymph gland. These tissues are mainstays of *Drosophila* developmental biologists, and so a unique GAL4-expressing line is highly prized because it can identify and define cell populations (through *UAS*-based reporter gene expression) within these tissues that may not be identifiable by any other genetic marker or structural feature. In total, students identified 563 different lines with developmental GAL4 expression in one or more of the tissues analyzed.

The use of the G-TRACE system by URCFG students for this analysis was particularly valuable because it allowed for GAL4 expression patterns (cell populations) to be analyzed on the basis of lineage marking in addition to the standard real-time patterns associated with traditional GAL4 expression screens. Accordingly, both the real-time expression (RFP) and lineage-traced expression (GFP) patterns were scored within each tissue analyzed (available online at the GED), which was particularly useful for identifying NP lines with dynamic GAL4 expression during development. As an example, the vast majority of the identified NP lines (21 of 25) that exhibit expression in the lobula primordial (LOP) of the optic lobe (highlighted above) show only GFP expression in the third instar, indicative of transient GAL4 activity during earlier developmental stages. Such lines would not have been identified with a standard real-time GAL4-expression analysis at the third instar. Future refined analyses of G-TRACE lineage patterns by advanced students or others in the research community may provide insight into the developmental relationship between progenitor and extant cell populations that may not be easily gained through other means. In addition to functioning as genetic reporters that define cell populations, GAL4 lines can also shed light upon the relationship of cells within tissues by serving as tools to alter gene function (*e.g.*, GAL4-mediated RNA interference).

Another scientific goal of the G-TRACE project was having URCFG students conduct basic bioinformatics analyses to associate endogenous gene expression with the patterns they observed within the tissues analyzed. This was an important exercise because understanding the genetic control of tissue development is a fundamental problem for biologists, and this provided a mechanism for identifying potentially biologically relevant genes while reinforcing the “discovery” educational experience. As described above, this study identified 1,031 genes that are potentially associated with the development of the brain, eye and wing imaginal discs, and the lymph gland. Within this set of genes, 421 were found to be uncharacterized, thereby opening the door for future exploration. Additionally, of the 537 NP lines exhibiting GAL4 expression, 340 (63%) were associated with a single proximal gene. Although we did not seek to further associate specific GAL4 expression with that of the corresponding candidate genes identified by this “gene discovery” approach, it follows that *Drosophila* researchers interested in more detailed analyses could easily perform such validations (*e.g.*, by *in situ* hybridization, RT-PCR, or immunostaining). Furthermore, the functional role of candidate genes expressed within the developing tissues could be explored through standard forward or reverse genetic analyses.

In addition to providing a modern, state-of-the-art research laboratory experience to undergraduates, a major goal of the G-TRACE project was to use it as a vehicle to bolster science education. A major hurdle of the science education laboratory is to implement a project in which students can feel a sense of ownership and scientific contribution, and can understand how their work fits in with the “big picture”. We have found in our previous large-scale research projects involving undergraduates that, without these elements, it is not uncommon for students to devalue their contribution and lose interest in the research. With the G-TRACE system, students quickly appreciated that they acquired their own results and that each “positive” they discovered (a line showing RFP and/or GFP expression patterns) was a unique contribution to the overall project and to the larger *Drosophila* research community. Screening success (*i.e.*, finding “positive” lines) on the part of URCFG students was facilitated by the use of lines pre-selected for some basal GAL4 activity (Hayashi *et al.* 2002), the evaluation of several different tissues, and the use of fluorescent reporter proteins in the G-TRACE system (Evans *et al.* 2009), which allowed each student to examine a greater number of GAL4-expressing lines than would otherwise be possible with non-fluorescent reporter proteins (*e.g.*, *lacZ*). The combination of these factors essentially assured the discovery of multiple interesting, if not novel, expression patterns by each student.

**Figure 7:**
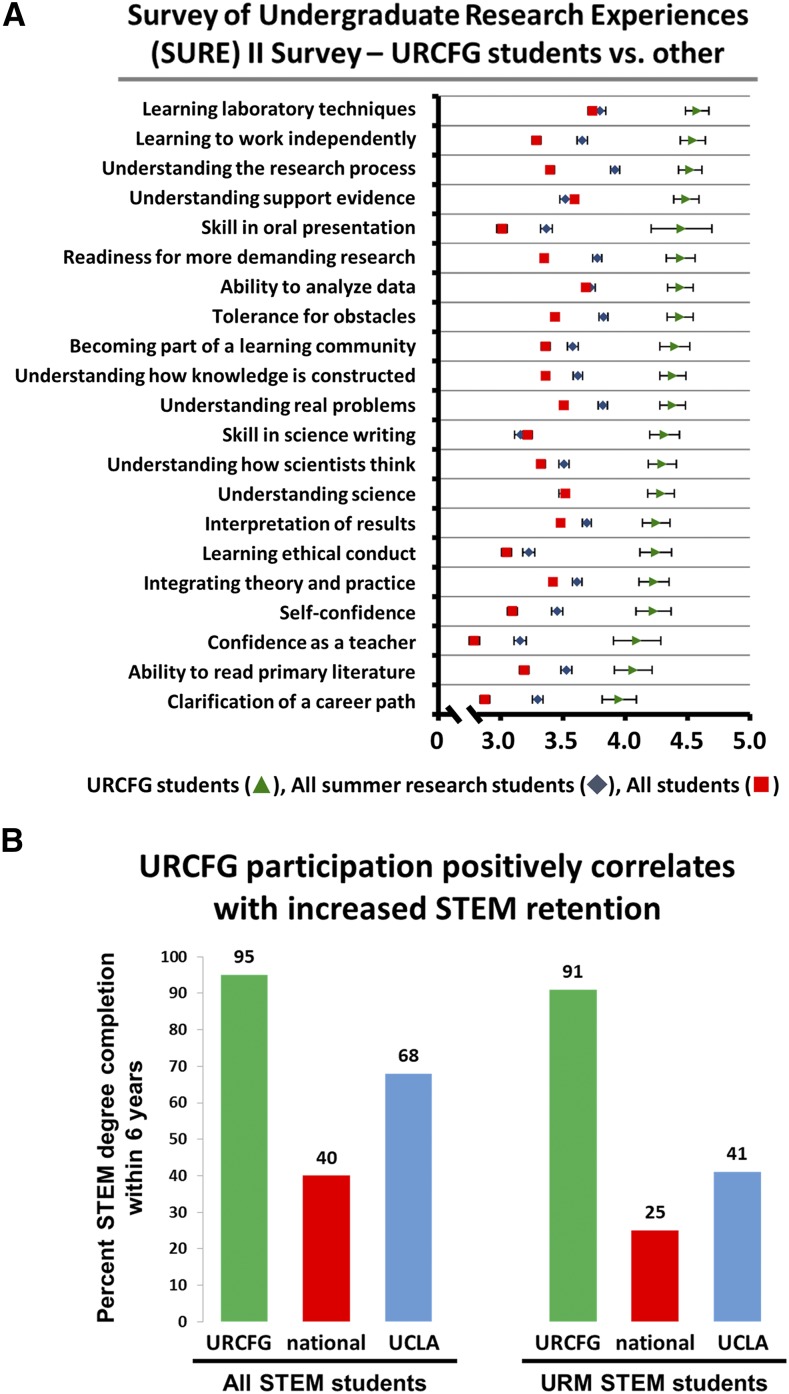
Impact of the URCFG experience on learning gains and STEM retention. A) Categorical data plot comparing reported learning gains between URCFG students (green triangles), students, nationally, completing summer research apprenticeships (All summer research students; blue diamonds), and students, nationally, completing introductory to advanced biology courses containing some research component (All students; red squares). Students participating in the URCFG exhibited increased gains across 21 different areas compared to students in the other groups. Learning gains were assessed using the Survey of Undergraduate Research Experiences (SURE) II, which offers both the Classroom Undergraduate Research Experiences (CURE) survey and the Summer Undergraduate Research Experience (SURE) survey. The CURE and SURE surveys include identical items that permit comparisons; URCFG students and “All students” took the CURE survey, while “All summer research students” took the SURE survey. The typical student in SURE cohorts was a third- or fourth-year student, and we compared to SURE 2013. Scale: 1 = little to no gain; 2 = small gain; 3 = moderate gain; 4 = large gain; 5 = very large gain. Error bars represent two times the standard error, representing greater than a 95% confidence interval. B) STEM retention rates are higher among URCFG students compared to national and UCLA averages. Degree completion data (6-year) is based on students enrolled in our URCFG CURE course from Winter 2003 through Spring 2018 (overall, n = 626; URM, n = 46). UCLA data were obtained from the Office of Analysis and Information Management (overall, n = 8,388; URM, n = 1,312). National data were obtained from Hurtado *et al.* (2012) (overall, n = 56,499; URM, n = 9,718). URCFG, Undergraduate Research Consortium for Functional Genomics; STEM, Science, Technology, Engineering, and Mathematics; URM, underrepresented minority.

The educational impact of our G-TRACE URCFG research program was captured by the Survey of Undergraduate Research Experiences (SURE) II survey, a quantitative method for assessing the benefits of undergraduate research (Lopatto 2004). URCFG students (conducting research over a single 10-week academic quarter) who took the survey reported greater learning gains in 21 different evaluative areas than students elsewhere participating in a full-time summer research or courses with a research component (Figure 7A).

The impact of the URCFG experience on the retention of undergraduate students in science, technology, engineering and mathematics (STEM) majors was also evaluated. Nationally, the STEM retention rate through degree completion has been estimated to be 40%, and drops to as low as 25% among underrepresented minority (URM) students (Hurtado *et al.* 2009, 2012; National Academies 2011; PCAST 2012). For comparison of URCFG student rates to UCLA and national benchmarks, we focused on 6-year degree completion rates in STEM disciplines. We analyzed UCLA Registrar data encompassing 46 academic quarters (Winter 2003 through Spring 2018) and found that 95% of URCFG STEM majors (n = 626) had completed a STEM degree within 6 years of enrollment at UCLA, more than twice the national average and considerably more than the UCLA average of 68% (Figure 7B). Importantly, STEM retention for URM students in the URCFG was just as high as that for non-URM students; 91% of URM STEM majors in the URCFG (n = 46) completed a STEM degree within 6 years, reflecting a rate of STEM retention nearly four times the national average and over twice the UCLA average of 41% (Figure 7B).

STEM retention, and in particular the retention gap between URM and non-URM students, is a persistent problem in higher education, even at highly selective universities such as UCLA. Because of this, the President’s Council of Advisors on Science and Technology has previously recommended that standard undergraduate laboratory courses be substituted with inquiry-based research courses (also known as Course-based Undergraduate Research Experiences or CUREs) as a means of increasing STEM retention (PCAST 2012). The data reported here points to the effectiveness of CURE pedagogical approaches in reducing or, in our case, eliminating the URM retention gap, and is consistent with previous studies that have shown that undergraduate research experiences, in the form of course-based research or laboratory apprenticeships, can improve STEM retention, especially for URM students (Nagda *et al.* 1998; Barlow and Villarejo 2004; Carter *et al.* 2009; Jones *et al.* 2010; Rodenbusch *et al.* 2016). Collectively, our findings, along with those of others, suggest that the opportunity to participate in a hands-on, active learning pedagogical approach is an important variable in promoting student learning and STEM retention, independent of institution type, overall student achievement, or socioeconomic background. Providing such opportunities to increasing numbers of early-stage undergraduate and high school students, particularly from underrepresented backgrounds, will be an important focus for future efforts.
